# Role of CD44 in increasing the potency of mesenchymal stem cell extracellular vesicles by hyaluronic acid in severe pneumonia

**DOI:** 10.1186/s13287-021-02329-2

**Published:** 2021-05-20

**Authors:** Li Zhou, Qi Hao, Shinji Sugita, Yoshifumi Naito, Hongli He, Che-chung Yeh, Jae-Woo Lee

**Affiliations:** 1 Department of Pulmonary and Critical Care Medicine, The Second Xiangya Hospital, Central South University, Changsha, Hunan China; 2 Department of Anesthesiology, University of California, San Francisco, 505 Parnassus Ave., Box 0648, San Francisco, CA USA

**Keywords:** CD44, Extracellular vesicles, Hyaluronic acid, Mesenchymal stem cell, *Pseudomonas aeruginosa* pneumonia

## Abstract

**Background:**

Although promising, clinical translation of human mesenchymal stem or stromal cell-derived extracellular vesicles (MSC EV) for acute lung injury is potentially limited by significant production costs. The current study was performed to determine whether pretreatment of MSC EV with high molecular weight hyaluronic acid (HMW HA) would increase the therapeutic potency of MSC EV in severe bacterial pneumonia.

**Methods:**

In vitro experiments were performed to determine the binding affinity of HMW HA to MSC EV and its uptake by human monocytes, and whether HMW HA primed MSC EV would increase bacterial phagocytosis by the monocytes. In addition, the role of CD44 receptor on MSC EV in the therapeutic effects of HMW HA primed MSC EV were investigated. In *Pseudomonas aeruginosa* (PA) pneumonia in mice, MSC EV primed with or without HMW HA were instilled intravenously 4 h after injury. After 24 h, the bronchoalveolar lavage fluid, blood, and lungs were analyzed for levels of bacteria, inflammation, MSC EV trafficking, and lung pathology.

**Results:**

MSC EV bound preferentially to HMW HA at a molecular weight of 1.0 MDa compared with HA with a molecular weight of 40 KDa or 1.5 MDa. HMW HA primed MSC EV further increased MSC EV uptake and bacterial phagocytosis by monocytes compared to treatment with MSC EV alone. In PA pneumonia in mice, instillation of HMW HA primed MSC EV further reduced inflammation and decreased the bacterial load by enhancing the trafficking of MSC EV to the injured alveolus. CD44 siRNA pretreatment of MSC EV prior to incubation with HMW HA eliminated its trafficking to the alveolus and therapeutic effects.

**Conclusions:**

HMW HA primed MSC EV significantly increased the potency of MSC EV in PA pneumonia in part by enhancing the trafficking of MSC EV to the sites of inflammation via the CD44 receptor on MSC EV which was associated with increased antimicrobial activity.

**Supplementary Information:**

The online version contains supplementary material available at 10.1186/s13287-021-02329-2.

## Background

*Pseudomonas aeruginosa* (PA) is the most frequent virulent gram-negative opportunistic pathogen causing community-acquired pneumonia among patients with chronic lung disease, older age, and immunocompromised status [1, 2], and it is often encountered in critically ill patients with respiratory failure [2–4]. However, due to the formation of a complex biofilm and existence of a wide variety of virulence factors, PA pneumonia is characterized by a high prevalence of multidrug resistance [1], increasing morbidity and mortality [5, 6]. Therefore, new innovative therapies are needed.

Cell-based therapy with mesenchymal stem or stromal cell (MSC) has emerged as an effective treatment for acute lung injury (ALI) in pre-clinical models [7]. However, potential limitations of cell-based therapy exist such as the risk of iatrogenic tumor formation and the significant cost for cell storage in a bone marrow transplant facility, limiting the number of patients who may benefit. To overcome these obstacles, we recently demonstrated that human bone marrow-derived MSC extracellular vesicles (EV) shared similar therapeutic properties as MSC in treating ALI in small animal models as well as the ex vivo perfused human lung injured with bacterial pneumonia [8–11]. MSC EV are membrane bound, anuclear fragments of cells, approximately 50–1000 nm in size, constitutively shed from the plasma membrane as microvesicles or released from the endosomal compartment as exosomes. MSC EV have the same functional phenotype as MSC such as the ability to home to the sites of inflammation and transfer mRNAs, microRNAs, proteins, lipids, and organelles with reparative and anti-inflammatory properties to injured cells. However, the number of MSC needed to generate enough EV for a similar response as MSC is approximately × 5–10 more [11, 12]. Therefore, it is crucial to develop methods to increase the potency of MSC EV.

Hyaluronic acid (HA) is a non-sulfated glycosaminoglycan, consisting of repeating polymeric disaccharides, D-glucuronic acid and N-acetyl-D-glucosamine, which is critical for maintaining the integrity of the extracellular matrix and homeostasis in the lung. Surprisingly, HA is involved in both tissue injury and repair depending on its molecular weight. HMW HA (≥ 1.0 MDa) enhances the secretion of anti-inflammatory cytokines by target cells, maintains both endothelial and epithelial barrier integrity, and promotes regeneration and repair of tissues [13], whereas low molecular weight HA (LMW HA) (< 500KDa), released as a degradation product during inflammation, induces inflammatory response through regulation of HA binding proteins such as CD44, TLR2 and 4, HABP2, or RHAMM [14, 15]. CD44 is a widely expressed cell-surface glycoprotein on immune cells such as alveolar macrophages and plays a critical role in the adhesion, uptake, and internalization of HA by these cells during inflammation [16, 17]. CD44 is also significantly expressed on MSC EV [18]. In the current proposal, we hypothesized that HMW HA primed MSC EV would increase the trafficking of the EV to the injured alveolus in severe PA pneumonia, increasing the overall interaction of MSC EV with target cells in part through CD44 expression on the EV.

## Methods

### Human bone marrow-derived MSC

Human bone marrow-derived MSC were obtained from a National Institutes of Health repository at Texas A&M Health Science Center, which fulfill the criteria for MSC as defined by the International Society of Cellular Therapy. MSC were cultured in Minimal Essential Medium alpha without nucleosides containing 16.5% fetal bovine serum, 0.2 M L-glutamine, and 1% penicillin-streptomycin. To obtain MSC EV, confluent MSC were cultured in condition medium (deprived of fetal bovine serum and supplemented with 0.5% bovine serum albumin) for 48 h as previously described [8]. The viability and quantity of serum-starved MSC were evaluated by trypan blue exclusion using hemacytometer. The supernatants from MSC were centrifuged at 3000 rpm for 20 mins to remove cellular debris and then underwent ultracentrifugation at 100,000*g* at 4 °C for 1 h twice. The pellets were re-suspended and washed with phosphate-buffered saline in between ultracentrifugation. MSC EV were re-suspended according to the final cell counts after 48 h serum starvation (10 μl of EV were equivalent to the vesicles released by 1 × 10^6^ cells over 48 h) and stored at – 80 °C. The size and number of MSC EV were analyzed using NanoSight NS300 (Malvern, Inc.). We previously characterized MSC EV using the current isolation technique [8, 11] as recommended by the International Society of Extracellular Vesicles [19].

### Binding ability of different molecular weights of HA to MSC EV

Glass slides were coated with PBS or LMW (40 KDa), HMW (1 MDa), or HMW (1.5 MDa) HA at a concentration of 10 mg/ml and dried in room temperature overnight [20, 21]. PKH26-labeled MSC EV (50 μl) were then added onto the chamber slides. Two hours later, chamber slides were washed extensively with PBS to remove unbound MSC EV. The slides were examined under fluorescence microscopy, and the intensity of fluorescent areas was analyzed using Image J software. MSC EV were labeled with PKH26 according to the manufacturer’s protocol, washed with PBS, and subjected to the same ultracentrifugation steps to isolate the EV. For the PBS control, the same volume of PBS as MSC EV was incubated with PKH26 and subjected to the same washing and ultracentrifugation steps to demonstrate that the ultracentrifugation step removed free PKH26.

### Uptake of MSC EV preincubated with different doses or molecular weights of HA by LPS stimulated human blood monocytes

Human blood monocytes were collected from fresh whole blood from healthy donors as previously described [20]. Monocytes were re-suspended in RPMI 1640 medium with 10% FBS and plated in 4-well chamber slides (5 × 10^5^ cells/well) at 37 °C in 5% CO_2_ incubator overnight. In order to compare the differences in uptake of MSC EV preincubated with different doses of HMW HA (1 MDa) by LPS stimulated monocytes, the cells were exposed to LPS (1 μg/ml) and (a) MSC EV (50 μl), (b) MSC EV + 0.2 μg/ml HMW HA (1.0 MDa), (c) MSC EV + 1 μg/ml HMW HA (1.0 MDa), or (d) MSC EV + 5 μg/ml HMW HA (1.0 MDa). MSC EV were labeled with PKH67 before incubation with HMW HA. MSC EV were then incubated with HA for 30 min prior to administration. After 24 h, the monocytes were stained with mounting media and DAPI (Vectashield, Vector Laboratories, CA). The uptake of MSC EV by monocytes was measured using fluorescence microscopy. In separate experiments, to determine the effect of different molecular weights of HA on the uptake of MSC EV by LPS stimulated monocytes, the monocytes were exposed to LPS and (a) MSC EV (50 μl), (b) LMW HA primed MSC EV [MSC EV preincubated with LMWHA (40 KDa)], (c) HMW HA(1.0 MDa) primed MSC EV [MSC EV preincubated with HMW HA (1.0 MDa)], or (d) HMW HA (1.5 MDa) primed MSC EV [MSC EV preincubated with HMW HA (1.5 MDa)]. The slides were fixed in 4% paraformaldehyde, and the uptake of MSC EV by human monocytes was measured using fluorescence microscopy. In all experiments described, MSC EV were incubated with HA for 30 min at 37 °C before administration onto cells or into mice.

### Phagocytosis of PA103 bacteria by LPS stimulated monocytes treated with HA primed MSC EV

Human blood monocytes were exposed to LPS (1 μg/ml) and (a) PBS, (b) MSC EV (90 μl), (c) LMW HA (40 KDa) primed MSC EV at a dose of 5 μg/ml or 0.5 μg total, (d) HMW HA (1.0 MDa) primed MSC EV, or (e) HMW HA (1.5 MDa) primed MSC EV for 24 h. Opsonized PA103 bacteria (10^7^ colony forming units (CFU)) were then added into each well and incubated at 37 °C for 90 min. Aliquots of the culture medium, serially diluted, were removed and plated on 5% sheep blood agar plates to count PA103 CFU levels in the supernatant. In separate experiments, the monocytes were injured with opsonized PA103 bacteria and incubated at 37 °C for 60 min. Gentamycin was then added to the culture medium. After 30 min, the monocytes were washed with D-PBS twice, and 1% Triton X-100 was added into each well. The cell lysates were collected and plated on 5% sheep blood agar plates with serial dilution to count the intracellular PA103 CFU load.

In separate experiments, the impact of CD44 siRNA pretreatment of MSC EV prior to incubation with HMW HA on bacterial phagocytosis by monocytes was investigated. MSC EV (90 μl) were transfected with CD44 siRNA or scrabbled siRNA (Negative Control No.1 siRNA, Ambion) for 24 h. MSC EV were washed extensively with PBS and isolated by ultracentrifugation. Then, human monocytes were exposed to LPS (1 μg/ml) and (a) PBS, (b) CD44 siRNA transfected MSC EV + HMW HA (1.0 MDa), or (c) scrabbled siRNA transfected MSC EV + HMW HA (1.0 MDa) for 24 h. Intracellular and extracellular PA 103 CFU levels were then measured.

### Severe PA103 pneumonia in mice

The Institutional Animal Care and Use Committee at the University of California San Francisco approved all experimental protocols. Male C57BL/6 mice (8–12 weeks of age, ~ 25 g; Jackson Laboratory, Bar Harbor, ME) were used in all the experiments. Mice were anesthetized with isoflurane, and then PA103 bacteria was instilled intratracheally (5 × 10^4^ CFU per mice). Four hours later, various treatments were instilled through the retro-orbital vein: (1) PBS as a carrier control, (2) MSC EV (90 μl), (3) HMW HA (1.0 MDa, dose 5 μg/ml or 0.5 μg total) primed MSC EV, (4) HMW HA alone (1.0 MDa, dose 5 μg/ml), (5) MSC (5 × 10^5^ cells/mouse), or (6) MSC preincubated with HMW HA (1.0 MDa, dose, 5 μg/ml). Mice were euthanized after 24 h, and the blood and bronchoalveolar lavage fluid (BALF) were collected for assessment of cell counts, bacterial CFU levels, inflammatory cytokines, and histology. In separate experiments, additional groups were studied: (1) CD44 siRNA transfected MSC EV + HMW HA (1.0 MDa, dose 5 μg/ml or 0.5 μg total), (2) scrabbled siRNA transfected MSC EV + HMW HA (1.0 MDa, dose 5 μg/ml or 0.5 μg total). The total cell counts and differential were measured by using the Hemavet HV950FS (Drew Scientific, Miami Lakes, FL). Mouse TNFα and IL-6 levels in the BALF and plasma were measured using ELISA kits (R&D Systems, Minneapolis, MN). For histology, mice lungs were fixed in 10% formalin and embedded in paraffin, cut into 4 μm sections, and stained with H&E. Levels of lung injury were determined by using semi-quantitative lung injury score as previously described [8]. For each mouse, 20 fields of the left lung at × 20 magnification were examined. Scoring was performed by grading as follows: infiltration or aggregation of inflammatory cells in air space or vessel wall: 1 = only wall, 2 = few cells (1–5 cells) in air space, 3 = intermediate, 4 = severe (air space congested); interstitial congestion and hyaline membrane formation: 1 = normal lung, 2 = moderate (50% of lung section); hemorrhage: 0 = absent, 1 = present.

### Trafficking of MSC EV in mice with PA103 pneumonia

Male C57BL/6 mice were intratracheally injured with PA103 bacteria (5 × 10^4^ CFU per mice). Four hours later, PKH26-labeled MSC EV (90 μl) or HMW HA (1.0 MDa) primed MSC EV was intravenously instilled into the retro-orbital vein. Six hours later, mice were euthanized, and the lungs, liver, and spleen were harvested. The organs were washed twice with cold PBS and fixed in 4% paraformaldehyde for 24 h at 4 °C. The organs were embedded in O.C.T (Tissue-Teck O.C.T. Compound, Sakura Finetek) and immediately frozen at − 80 °C. Frozen 10-μm slices were stained with mounting media with DAPI (Vectashield). The intensity of PKH26-labeled MSC EV in the organs was measured using Cytation 5 cell imaging multi-mode reader (Bio Tek Instruments).

In separate experiments, the role of MSC EV CD44 expression in trafficking of HMW HA (1.0 MDa) primed MSC EV was studied. MSC EV were transfected with a CD44 siRNA or scrabbled control siRNA. PKH26-labeled CD44 or scrabbled siRNA-treated MSC EV (90 μl) incubated with HMW HA (1.0 MDa) were intravenously instilled 4 h following pneumonia. Six hours later, mice lungs, liver, and spleen were harvested, processed, and analyzed as before.

### Statistics analysis

Data were presented as the mean ± SD or median with interquartile range (IQR). Shapiro-Wilk normality test was used to determine if the values were from a Gaussian distribution. Comparisons between the two groups were made using Student’s *t* test or the Mann-Whitney *U* test. Comparisons between more than two groups were made using ANOVA with the Bonferroni’s correction or Kruskal-Wallis test with Dunn’s correction. *P* < 0.05 was considered statistically significant. Graphpad Prism 8 software was used for statistical analysis.

## Results

### Characterization of MSC EV and MSC EV binding to hyaluronic acid

**Fig. 1:**
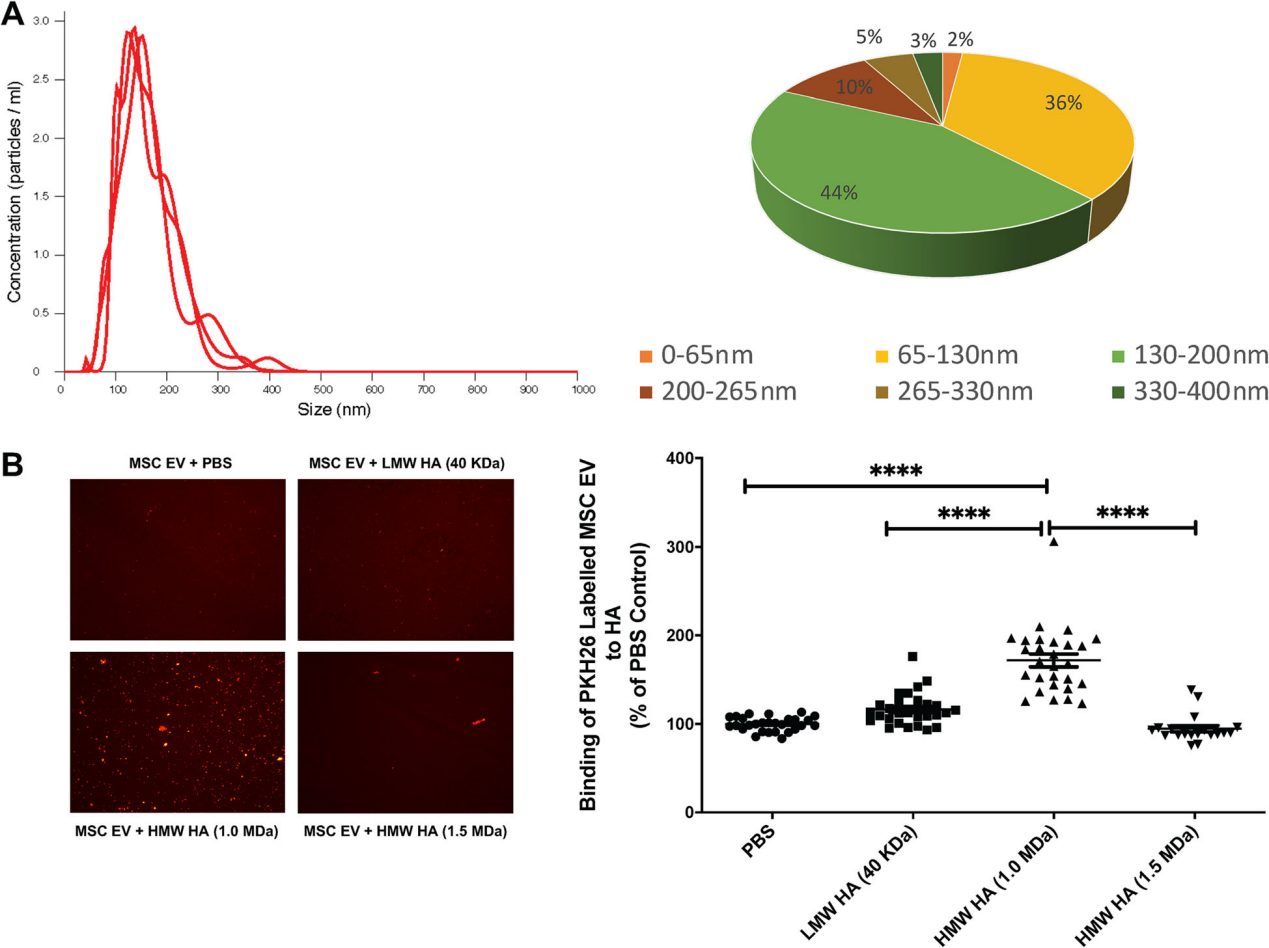
MSC-derived Extracellular Vesicles and Influence of Molecular Weight of Hyaluronic Acid on EV Binding. **a** Mean size and size distribution of MSC EV by NanoSight analyses. **b** Binding ability of different molecular weights of HA with MSC EV. PKH26-labeled MSC EV preferentially bound to HMW HA (1.0 MDa) compared with PBS, LMW HA (40 KDa), or HMW HA (1.5 MDa). Data are mean ± SD, ****P < 0.0001 by ANOVA (Bonferroni), *N* = 19–28. Dose of MSC EV (50 μl) used = 1.1 × 10^10^ particles

**Fig. 2:**
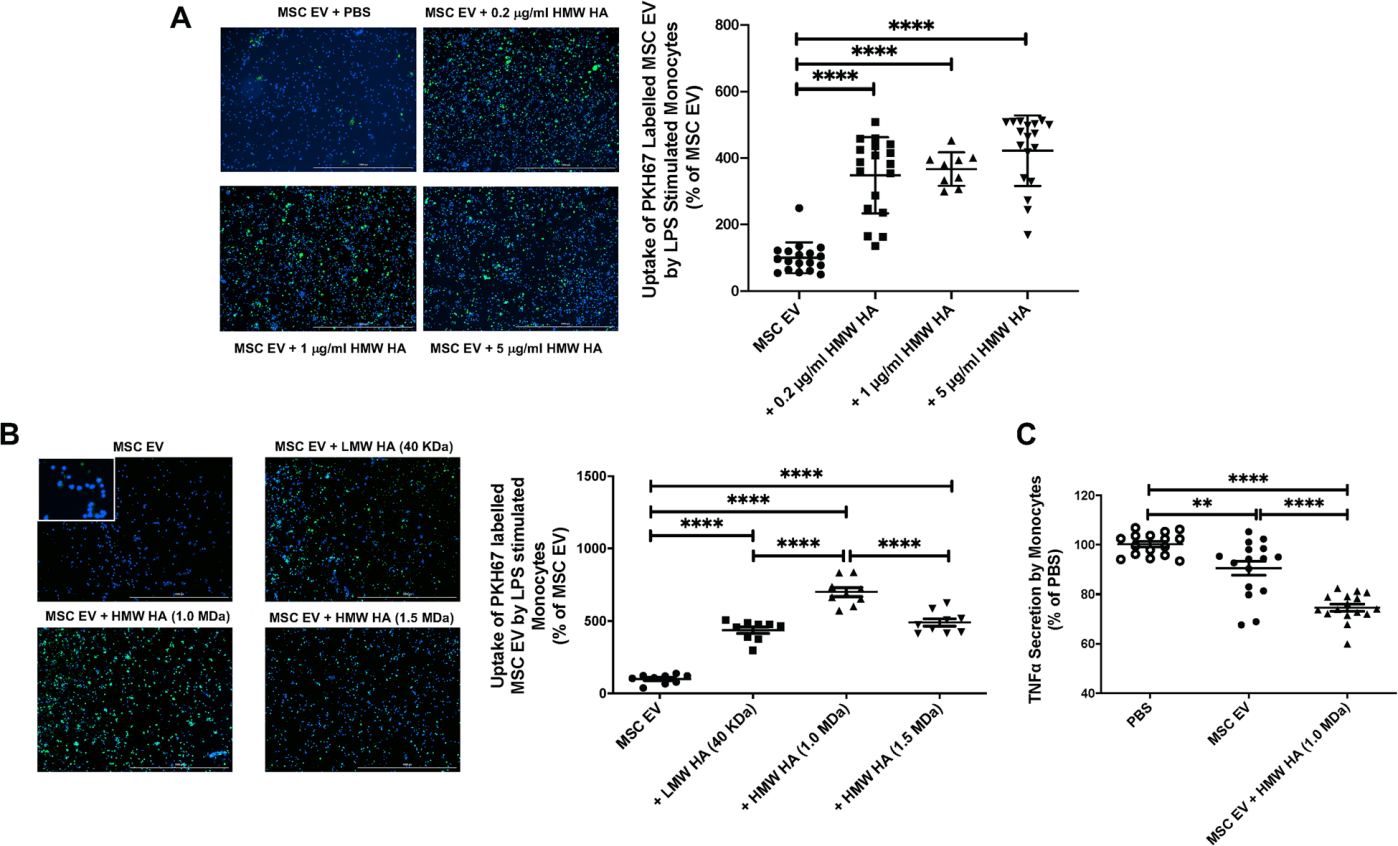
Effect of Different Molecular Weights of Hyaluronic Acid on MSC EV Uptake by Human Monocytes. **a** Effect of pre-incubation of MSC EV with different doses of HMW HA (1.0 MDa) on its uptake by LPS stimulated human monocytes. Compared with MSC EV alone, MSC EV pre-incubated with HA doses of 0.2, 1, or 5 μg/ml HMW HA (1.0 MDa) showed increased uptake by LPS stimulated human monocytes. There were no significant differences between the three doses of HMW HA. Data are mean ± SD for each condition, ****P < 0.0001 by ANOVA (Bonferroni), *N* = 9–18. Bar = 1 mM. **b** Effect of MSC EV pre-incubated with different molecular weights of HA on MSC EV uptake by LPS stimulated monocytes. LMW HA (40 KDa) primed MSC EV, HMW HA (1.0 MDa) primed MSC EV, and HMW HA (1.5 MDa) primed MSC EV all showed increased uptake by LPS stimulated monocytes when compared with MSC EV alone. HMW HA (1.0 MDa) primed MSC EV demonstrated the highest uptake by the monocytes. Data are mean ± SD, ****P < 0.0001 by ANOVA (Bonferroni), *N* = 9. Bar = 1 mM. **c** HMW HA (1.0 MDa) primed MSC EV significantly decreased the secretion of TNFα by LPS stimulated human monocytes when compared with MSC EV alone. Data are mean ± SD, **P < 0.01, ****P < 0.0001 by ANOVA (Bonferroni), *N* = 16. Dose of MSC EV (50 μl) used = 1.1 × 10^10^ particles

The mean size and concentration of MSC EV were 164.9 ± 1.4 nm and 2.2 ± 0.02 × 10^11^ particles/ml respectively (Fig. 1a). Fluorescence microscopy showed that MSC EV bound preferentially to HMW HA (1.0 MDa) compared with LMW HA (40 KDa) or, surprisingly, HMW HA (1.5 MDa) (Fig. 1b). Incubation with MSC EV with HMW HA (1.0 MDa) significantly increased its uptake by human monocytes compared to MSC EV alone at doses between 0.2 and 5 μg/ml (Fig. 2a). All subsequent experiments were performed with a HA dose of 5 μg/ml, which was the lowest dose of HMW HA which would both significantly bind MSC EV but also have a biological effect when administered to injured monocytes as HMW HA primed MSC EV.

### Effect of preincubation of MSC EV with different molecular weights of HA on its uptake by LPS stimulated human monocytes

HMW HA (1.0 MDa) primed MSC EV showed the most significant uptake by LPS stimulated monocytes compared to incubation with LMW or HMW (1.5 MDa) HA (Fig. 2b). In addition, treatment with HMW HA (1.0 MDa) primed MSC EV further suppressed the secretion of TNFα compared with control PBS or MSC EV alone treatment in the medium of LPS stimulated human monocytes (Fig. 2c).

### Effect of HMW HA primed MSC EV on the phagocytosis of PA103 bacteria by LPS stimulated human monocytes

**Fig. 3:**
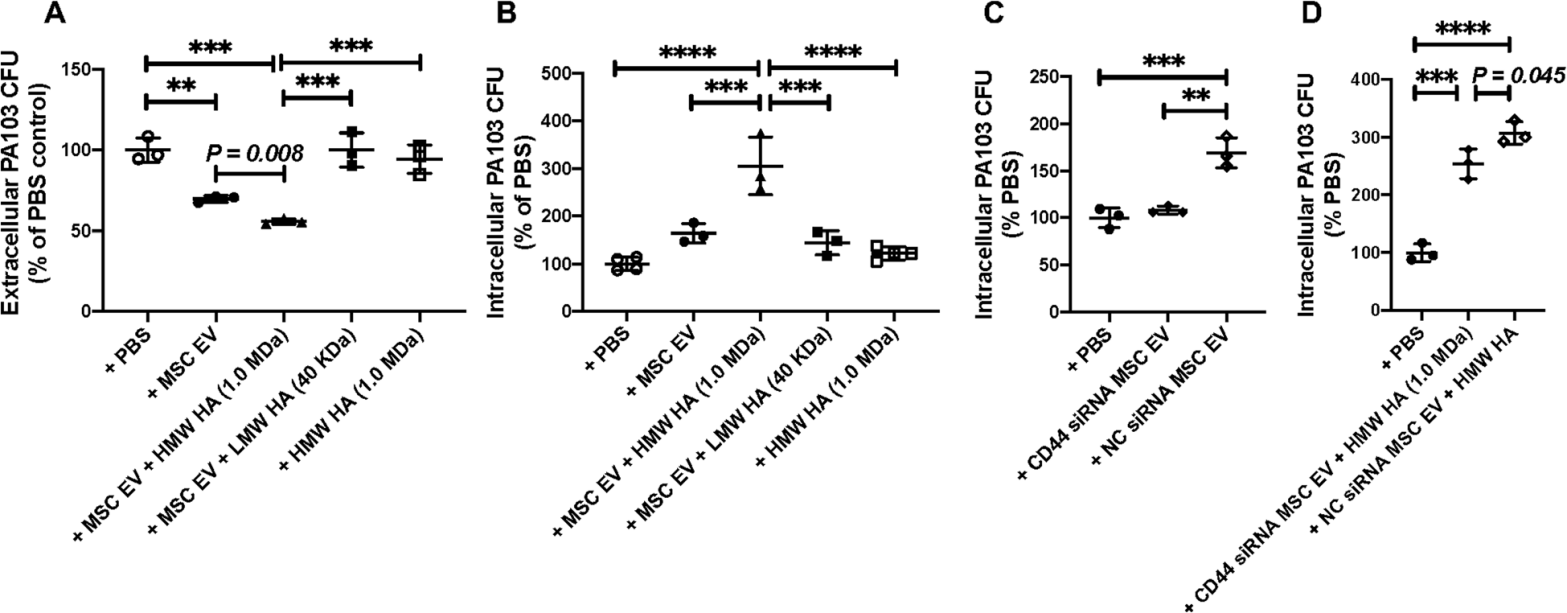
Effect of Different Molecular Weights of Hyaluronic Acid Primed MSC EV on PA103 Bacterial Phagocytosis. **a**, **b** Extracellular bacterial CFU levels in the culture medium and Intracellular bacterial CFU levels as measured in LPS stimulated monocytes when treated with different MW HA primed MSC EV. Administration of HMW HA (1.0 MDa) primed MSC EV reduced extracellular PA103 CFU and increased intracellular PA103 CFU levels when compared with MSC EV or LMW HA (40 KDa) primed MSC EV or HMW HA (1.0 MDa) alone. **c**, **d** Intracellular bacterial CFU levels of LPS stimulated monocytes when treated with CD44 siRNA pretreated MSC EV with or without HMW HA. CD44 siRNA pretreatment of MSC EV prior to HMW HA incubation decreased bacterial phagocytosis by human monocytes compared to negative control (NC) siRNA pretreated MSC EV. Data are mean ± SD, **P < 0.01, ***P < 0.001, ****P < 0.0001 by ANOVA (Bonferroni), *N* = 3. Individual *P* values describe the statistical comparison between the two groups by Student’s *t* test. A dose of MSC EV (90 μl) was used = 2.0 × 10^10^ particles

Incubation with HMW HA (1.0 MDa) primed MSC EV further increased PA bacterial phagocytosis by LPS stimulated human monocytes compared to PBS or MSC EV treatment (Fig. 3a, b). As controls, there was no therapeutic effect of HMW HA (1.0 MDa) alone on inflammation or bacterial phagocytosis of LPS stimulated human monocytes at the dose used for pre-incubation. Pretreatment of MSC EV with CD44 siRNA significantly reduced bacterial phagocytosis by human monocytes either with or without HMW HA co-incubation compared to scrabbled siRNA pretreated MSC EV (Fig. 3c, d).

### Therapeutic effects of HMW HA primed MSC EV in mice injured with severe PA103 bacterial pneumonia

**Fig. 4:**
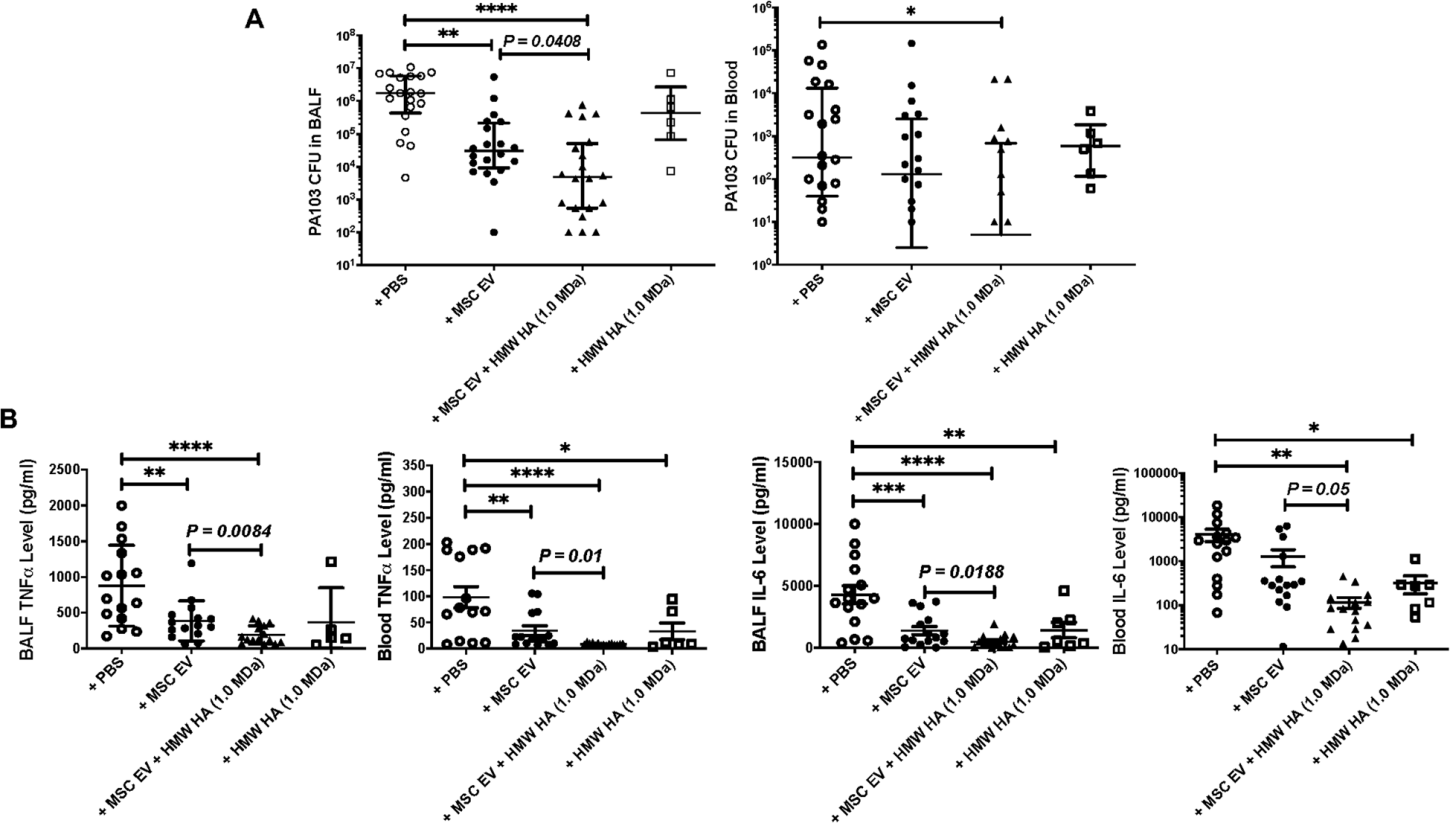
Therapeutic Effects of MSC EV Primed HMW HA in Mice Injured with PA103 Bacterial Pneumonia. **a** Intravenous administration of MSC EV significantly decreased PA103 CFU levels in the BALF and blood when compared with injured mice. Treatment of HMW HA (1.0 MDa) primed MSC EV further reduced the PA103 CFU levels in the BALF and blood compared with MSC EV treated mice. Data are median with IQR, *P < 0.01, **P < 0.01, ****P < 0.0001 by Kruskal-Wallis test with Dunn’s correction, *N* = 6–20. Individual *P* values describe the statistical comparison between the two groups by Mann-Whitney *U* test. **b** Administration of MSC EV primed with or without HMW HA (1.0 MDa) both significantly reduced TNFα and IL-6 levels in the BALF and plasma in mice injured with severe PA pneumonia compared with injured mice. Compared to MSC EV, HMW HA (1.0 MDa) primed MSC EV further decreased TNFα and IL-6 levels in the BALF and plasma. Data are as mean ± SD, *P < 0.05, **P < 0.01, ***P < 0.001, ****P < 0.0001 by ANOVA (Bonferroni), *N* = 5–15. Individual *P* values describe the statistical comparison between the two groups by Student’s *t* test. A dose of MSC EV (90 μl) was used = 2.0 × 10^10^ particles

Intratracheal instillation of PA103 bacteria induced severe but nonlethal ALI at 24 h in mice with a high bacterial load [data are median with IQR, *N* = 20, 1.5 × 10^6^ CFU/ml (1.8 × 10^5^ to 5.6 × 10^6^)] at 24 h. Intravenous administration of MSC EV at 4 h after injury as therapy significantly decreased the bacterial CFU levels in the BALF by 98% [N = 20, 3.1 × 10^4^ CFU/ml (9.3 × 10^3^ to 2.2 × 10^5^)] compared with injured mice. More importantly, PA103 CFU levels in the BALF among injured mice treated with HMW HA (1.0 MDa) primed MSC EV [N = 20, 4883 CFU/ml (550 to 5.1 × 10^4^)] further reduced the bacterial levels by an additional 84%. Treatment of mice with HMW HA (1.0 MDa) alone at 4 h following PA103 bacterial pneumonia had no significant effect on the total bacterial load at 24 h [*N* = 6: 4.4 × 10^5^ CFU/ml (6.7 × 10^4^ to 2.7 × 10^6^)] (Fig. 4a). In a similar fashion, intravenous administration of HMW HA (1.0 MDa) primed MSC significantly reduced the BALF PA103 CFU levels compared to injured mice treated with MSC alone (Supplementary Figure 1). Pretreatment of MSC EV with CD44 siRNA eliminated the therapeutic effects of HMW HA primed MSC EV on PA103 bacterial CFU level in the BALF compared to mice treated with scrabbled siRNA pretreated MSC EV (Supplementary Figure 2).

Intratracheal instillation of PA103 bacteria also resulted in elevated PA103 levels in the blood [data are median with IQR: 318 CFU/ml (40 to 1.3 × 10^4^)] of mice at 24 h. Intravenous administration with MSC EV at 4 h after injury numerically decreased the total blood bacterial load by 59% [data are median with IQR: 130 CFU/ml (3 to 2576)] although not statistically significant. Intravenous administration of HMW HA (1.0 MDa) primed MSC EV further decreased the bacteremia by 96% [data are median with IQR: 5 CFU/ml (0 to 693)] which was statistically significant compared with the injured group (Fig. 4a). Similarly, treatment with HMW HA (1.0 MDa) primed MSC further significantly decreased blood PA103 CFU levels compared with treatment with MSC alone (Supplementary Figure 1). Again, treatment of injured mice with HMW HA (1.0 MDa) alone [data are median with IQR: 598 CFU/ml (116 to 1853)] had no significant effect on blood bacterial levels. The bacteremia in injured mice treated with CD44 siRNA-pretreated HMW HA primed MSC EV were significantly higher compared with the injured mice treated with scrabbled siRNA pretreated HMW HA primed MSC EV (Supplementary Figure 2).

Intravenous administration of MSC EV at 4 h after injury significantly reduced TNFα levels in the BALF and plasma among the ALI mice by 56% and 76%, respectively, compared with the injured control. HMW HA (1.0 MDa) primed MSC EV further decreased TNFα level in the BALF and plasma by an additional 51% and 55% respectively compared with injured mice treated with MSC EV (Fig. 4b). Similarly, HMW HA (1.0 MDa) primed MSC further reduced BALF TNFα level compared to treatment with MSC (Supplementary Figure 1). Compared with the treatment of MSC EV, treatment of HMW HA (1.0 MDa) primed MSC EV further significantly reduced the IL-6 levels both in the BALF and serum by 65% and 91% respectively (Fig. 4b). TNFα levels in the BALF and plasma in mice treated with CD44 siRNA pretreated HMW HA primed MSC EV were significantly higher compared to the injured group treated with scrabbled siRNA pretreated HMW HA primed MSC EV (Supplementary Figure 2).

### Therapeutic effects of HMW HA primed MSC EV on lung inflammatory cell counts and histology in mice injured with severe PA103 pneumonia

**Fig. 5:**
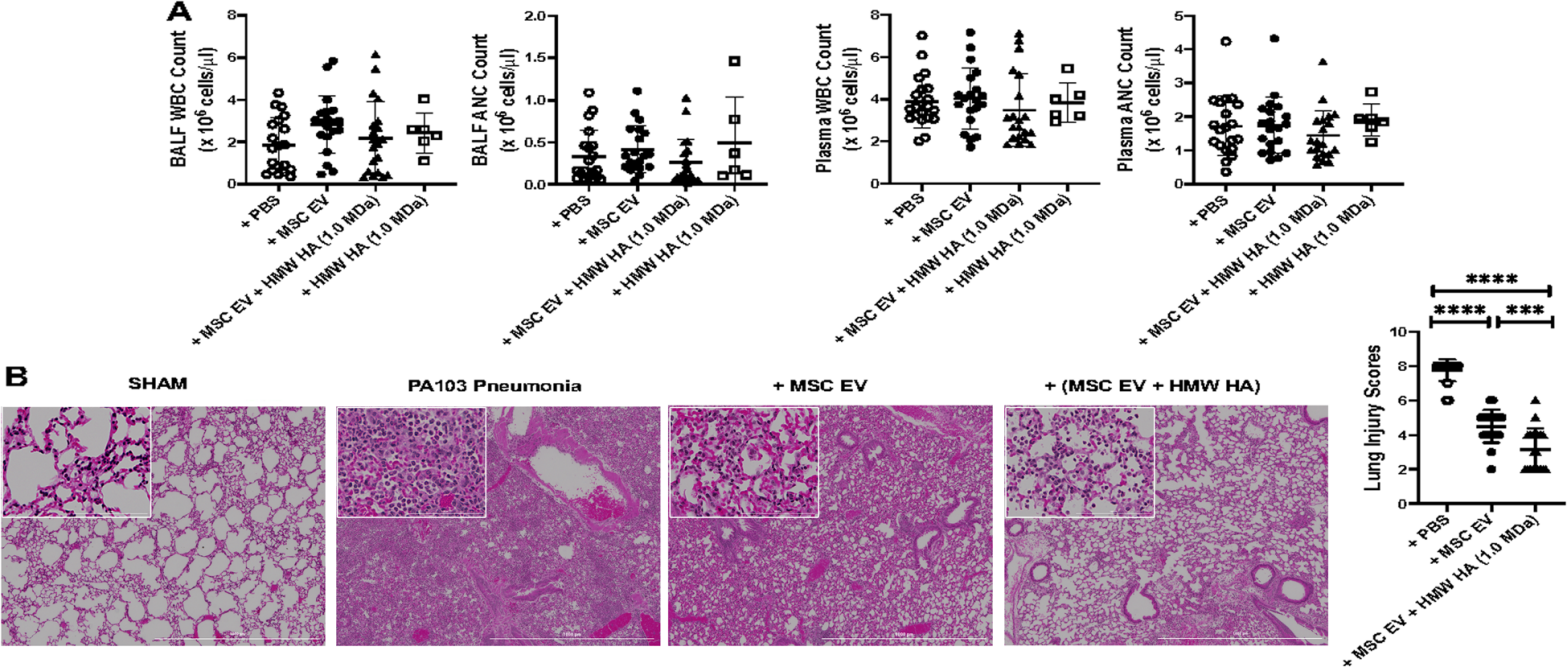
Effect of HMW HA Primed MSC EV on Inflammatory Cell Infiltration into the Injured Alveolus. **a** There were no significant differences in the total white blood cell (WBC) nor absolute neutrophil (ANC) counts in either the BALF or plasma. Data are mean ± SD, *N* = 6–20. **b** By histology, administration of MSC EV reduced alveolar inflammatory cells infiltration, interstitial wall thickening, and blood/edema, which was further reduced with HMW HA primed MSC EV. Lung injury assessed by semiquantitative scoring was decreased by MSC EV and further reduced with HMW HA primed MSC EV. Magnification × 4, Bar = 1 mM. Data are as mean ± SD, ***P < 0.001, ****P < 0.0001 by ANOVA (Bonferroni), *N* = 20. A dose of MSC EV (90 μl) was used = 2.0 × 10^10^ particles

There were no significant differences in total white blood cell or neutrophil counts between injured mice and the MSC EV treatment groups either in the BALF or serum (Fig. 5a). However, injured mice treated with MSC or HMW HA (1.0 MDa) primed MSC showed significantly reduced WBC and neutrophil counts in the plasma compared to the PBS treated group (Supplementary Figure 1). By histology, treatment with intravenous MSC EV 4 h after injury reduced the infiltration of inflammatory cells, edema, wall thickening, and airspace congestion at 24 h (Fig. 5b). Compared with the treatment of MSC EV, administration of HMW HA (1.0 MDa) primed MSC EV showed further improvement. In addition, CD44 siRNA pretreatment of MSC EV largely inhibited the therapeutic effects of HMW HA primed MSC EV on lung pathology (Supplementary Figure 2).

### Effect of HMW HA primed MSC EV on MSC EV trafficking to the lung, liver, and spleen

**Fig. 6:**
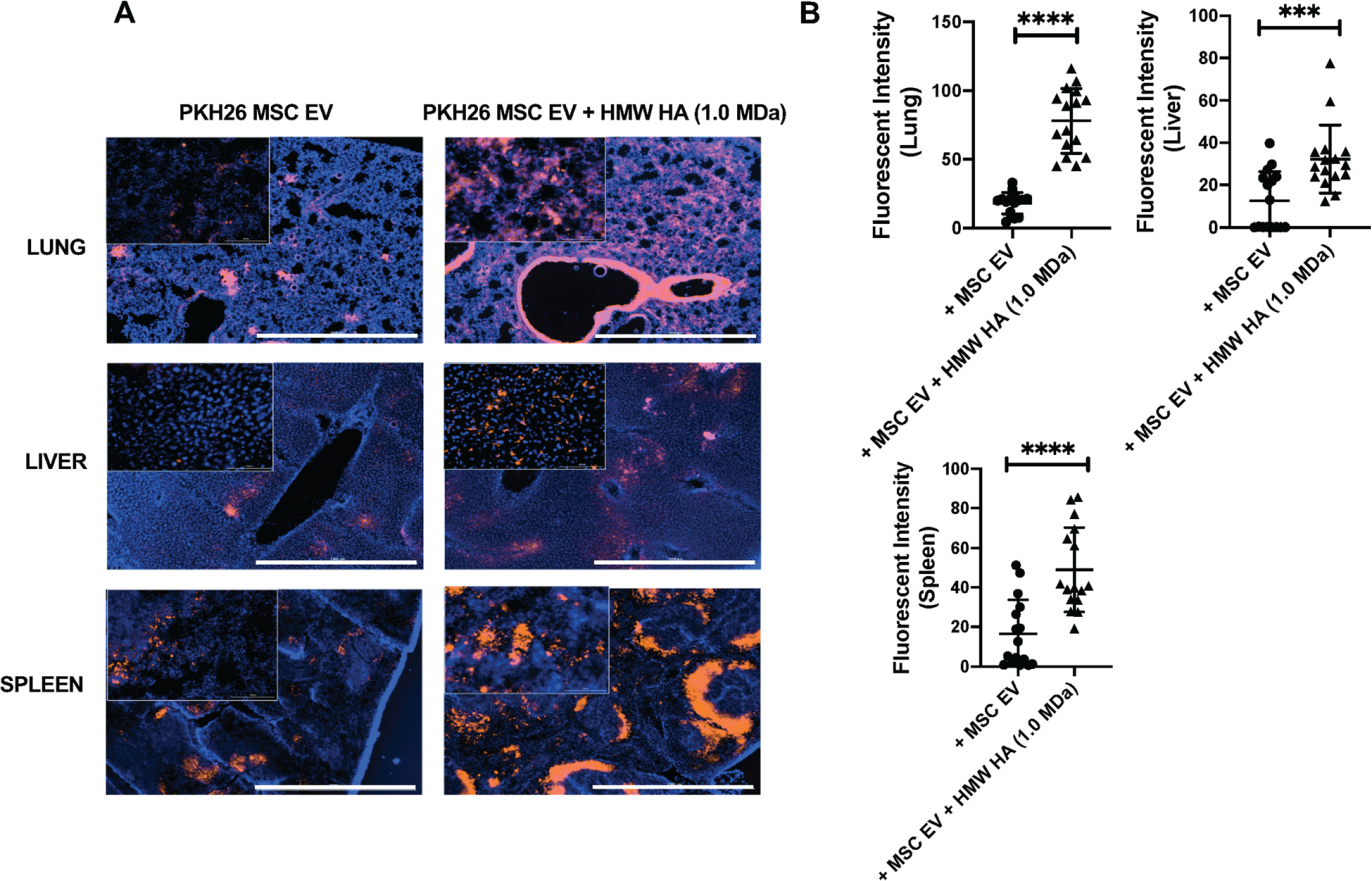
Trafficking of MSC EV With or Without HMW HA in Mice with PA103 Bacterial Pneumonia. Intravenous administration of HMW HA (1.0 MDa) primed MSC EV increased trafficking of MSC EV to the lung, liver, and spleen. **a** Representative immunofluorescence from each organ with treatment of HMW HA (1.0 MDa) primed MSC EV or MSC EV alone. Magnification 4X, Bar = 1 mM. **b** Quantification of the fluorescent intensity. Data is mean ± SD, ***P < 0.001, ****P < 0.0001 by Student’s *t* test, *N* = 12–16. A dose of MSC EV (90 μl) was used = 2.0 × 10^10^ particles

When compared with MSC EV, intravenous administration of MSC EV preincubated with HMW HA (1.0 MDa) showed increased trafficking of MSC EV to the injured pulmonary alveolus, liver, and spleen (Fig. 6). Administration of CD44 siRNA pretreated MSC EV + HMW HA demonstrated significantly decreased trafficking of MSC EV to the injured lung and liver compared to control scrabbled siRNA pretreated MSC EV + HMW HA (Supplementary Figure 3).

## Discussion

The main findings of the current study are (1) MSC EV bound preferentially to HMW HA (1.0 MDa) compared with LMW or HMW HA (1.5 MDa), which was associated with significantly higher uptake by LPS stimulated human monocytes and increased antimicrobial activity by the monocytes (Figs. 1, 2, and 3); (2) intravenous administration of MSC EV decreased PA103 CFU levels in the injured alveolus and blood in mice with severe PA103 bacterial pneumonia, which was further enhanced with HMW HA pre-incubation (Figs. 4 and 5); the antimicrobial effect of HMW HA primed MSC EV in the BALF and blood was significantly reduced when MSC EV was pretreated with CD44 siRNA (Supplementary Figure 2); (3) pre-incubation with HMW HA (1.0 MDa) enhanced the trafficking of MSC EV into sites of inflammation such as the lung, spleen, and liver (Fig. 6). However, CD44 siRNA pretreatment of MSC EV inhibited the increase in trafficking (Supplementary Figure 3).

**Fig. 7:**
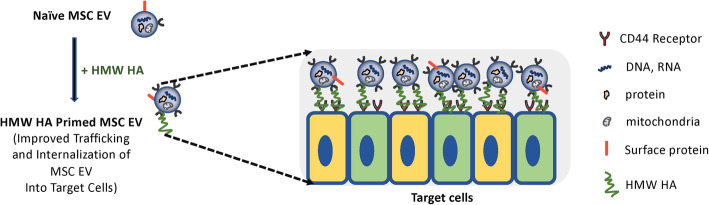
Schematic of Potential Mechanisms Underlying the Therapeutic Effects of HMW HA Primed MSC EV. By binding to CD44 on MSC EV, HMW HA further increased the trafficking, adhesion, and internalization of MSC EV into target cells in the injured alveolus, increasing the potency of the EV. We speculate that HMW HA may be a linker between MSC EV and target immune cells which leads to the internalization of the EV

The therapeutic use of MSC in preclinical models of ALI has shown great promise, leading to multiple phase I/II clinical trials despite some long-term concerns of cell-based therapy [22]. As a safe alternative to MSC, we and other investigators have found that administration of MSC EV ameliorated lung edema and protein permeability, alveolar inflammation, and decreased alveolar bacterial load in ALI from LPS or bacterial pneumonias [8, 9, 11, 23]. However, the major limitation for clinical translation of MSC EV is the decrease in potency compared to the cell, potentially making the production cost prohibitive for patients [24]. In the current study, we hypothesized that HMW HA primed MSC EV would increase EV trafficking to the site of injury and interaction with target cells, increasing the overall potency of MSC EV (Fig. 7).

We previously demonstrated that CD44 is a critical surface receptor on MSC EV mediating the uptake and internalization of the EV, which were important for its therapeutic response [8, 10]. CD44 is a trans-membrane glycoprotein highly expressed on multiple immune cells which is involved in regulating inflammatory response during ALI and infection [14, 16]. Moreover, the interaction between exposed HA in the extracellular matrix during ALI and CD44 on immune cells is also critical for the activation, recruitment, migration, extravasation, and homing of inflammatory cells [17, 25]. It is also known that CD44-HA signaling has important regulatory roles largely based on its MW in various pulmonary diseases such as bacterial pneumonia, LPS-induced lung injury, ventilator-induced lung injury, and asthma [14, 26]. In the current study, we found that MSC EV bound preferentially to HMW HA (1.0 MDa) compared to LMW HA (40 KDa) (Fig. 1). Previous studies have demonstrated that the affinity of HA-CD44 interaction was enhanced with the increase in MW of HA due to its multivalent property [17, 27, 28]. Jiang et al. showed a similar response in that the maximum binding affinity between HA and CD44 occurred at a MW of 1.0 MDa due to the balance between multivalent HA effect and conformational entropy [29]. In addition, HMW HA (1.0 MDa) primed MSC EV increased the uptake of EV by human monocytes, enhancing the therapeutic effects of EV on inflammation and bacterial phagocytosis by monocytes compared to MSC EV alone. We previously reported that MSC EV uptake by injured human monocytes was CD44 receptor-dependent, probably on the monocytes. Administration of MSC EV with an anti-CD44 Ab largely abolished the therapeutic effects of the EV in a severe pneumonia model in vivo [8]. In the current study, this effect was eliminated with CD44 siRNA pretreatment of the MSC EV (Fig. 3), indicating that CD44 plays an important role in the therapeutic effects of HMW HA primed MSC EV. CD44 expression on MSC EV itself may be critical for increased binding to HMW HA and the increased uptake of the EV by injured immune cells in the alveolus; HMW HA may be the linker between the target cells in the injured alveolus and the MSC EV which leads to the endocytosis of the EV (Fig. 7). In support of the hypothesis, CD44 siRNA pretreatment of MSC EV prior to incubation with HMW HA (1.0 MDa) inhibited the antimicrobial effect of MSC EV on injured human monocytes.

In a severe PA 103 pneumonia in mice, we demonstrated that intravenous administration of MSC EV decreased alveolar and systematic inflammation and increased bacterial clearance, which was further enhanced with HMW HA (1.0 MDa) primed MSC EV (Fig. 4) and which was again significantly reduced with CD44 siRNA pretreatment of the MSC EV (Supplementary Figure 2). A similar effect was seen with the administration of HMW HA primed MSC in terms of alveolar and systemic inflammation and bacterial load (Supplementary Figure 1). Administration of HMW HA (1.0 MDa) primed MSC EV showed increased trafficking of the EV to sites of inflammation (i.e., the lung, spleen and liver). Pretreatment of MSC EV with CD44 siRNA prior to incubation with HMW HA (1.0 MDa) largely eliminated the trafficking of the EV to the lungs and liver in mice with severe PA103 pneumonia (Fig. 6 and Supplementary Figure 3). A possible explanation may be that HMW HA attached to MSC EV further increased the trafficking and adhesion of EV to the injured alveolus which led to increased endocytosis of the EV by immune cells in the lungs (Fig. 7). These results were consistent with the study conducted by Corradetti et al. and Bian et al., in which they found that growing MSC on HA-coated tissue culture plates improve MSC homing towards inflammatory sites and demonstrated that HA facilitated the migratory ability of MSC to the injured kidney via CD44-HA interactions respectively [30, 31]. However, other mechanisms may account for the increase in trafficking. For example, studies are ongoing to determine if HMW HA itself may modify the target immune cells in the injured alveolus to increase MSC EV uptake by the cells.

In a previous study, we demonstrated that administration of exogenous HMW HA suppressed ALI in an ex vivo perfused human lung injured with severe *E. coli* pneumonia [20]; exogenous HMW HA has been studied in various ALI models (both sterile and infectious) as a therapeutic due to its immunomodulatory properties as well as ability to preserve endothelial barrier integrity. We speculated that HMW HA may bind to inflammatory EV released into the plasma and injured alveolus, preventing its interaction with target cells, which may be contradictory to our current hypothesis. However, the dose of HMW HA we used in the present study for pre-incubation with MSC EV was significantly smaller than in experiments with the ex vivo perfused human lung or even in mice with ALI from sepsis [32]. Thus, the dose of HMW HA may influence its behavior on MSC EV; too large of a dose may “sugarcoat” the MSC EV [33] and prevent its interaction with the target cells whereas a significantly smaller dose may increase the trafficking of the MSC EV to sites of inflammation.

There are some limitations to the current study: (1) although incubation with LMW HA (40 KDa) or HMW HA (1.5 MDa) did increase MSC EV uptake by LPS stimulated human monocytes, there were no effects on bacterial phagocytosis or inflammation. Studies are ongoing to determine if there were any differences in the binding kinetics with CD44 on human monocytes with different MW of HA which may affect phagocytosis; (2) despite the significant effect of MSC EV on bacterial phagocytosis by monocytes in vitro and alveolar inflammation and bacterial load following severe pneumonia in vivo, the target cells of MSC EV are currently unknown. Future studies are planned with fluorescence-activated single-cell sorting to isolate the MSC EV targeted cells in the lung.

## Conclusion

Administration of HMW HA (1.0 MDa) primed MSC EV further enhanced the therapeutic effects of MSC EV in terms of alveolar inflammation and bacterial load compared with MSC EV in mice injured with PA pneumonia, which was associated with increased trafficking to the sites of inflammation in the lung, liver, and spleen due to CD44 expression on the EV. HMW HA (1.0 MDa) primed MSC EV may be a promising method to increase the potency of MSC EV, addressing one of the major limitations for translation into clinical trials.

## Supplementary Information

**Additional file 1: Supplementary Figure 1.** Therapeutic Effects of MSC Pre-incubation with HMW HA in Mice Injured with PA103 Bacterial Pneumonia. (A) Intravenous administration of MSC with or without pre-incubation with HMW HA (1.0 MDa) significantly decreased the PA103 CFU levels in the BALF and blood. Data are median with IQR, *P < 0.05, **P < 0.01, ****P < 0.0001 by Kruskal Wallis test with Dunn’s Correction, *N* = 16–20. (B) Intravenous administration of MSC significantly reduced TNFα levels in the BALF compared to injured mice, which was further decreased with pre-incubation with HMW HA (1.0 MDa). Data are mean ± SD, *P < 0.05, ***P < 0.001 by ANOVA (Bonferroni), *N* = 14–15. (C) Compared to PBS, MSC pre-incubated with HMW HA (1.0 MDa) significantly reduced blood WBC and absolute neutrophil counts. Data are mean ± SD, *P < 0.05 by ANOVA (Bonferroni), N = 16–20. Individual *P* values describe the statistical comparison between the two groups by Mann Whitney U test or Student t-test.

**Additional file 2: Supplementary Figure 2.** Influence of CD44 siRNA Pretreatment of MSC EV in the Therapeutic Effects of HMW HA Primed MSC EV in Mice with PA103 Bacterial Pneumonia. (A) CD44 siRNA pretreatment of MSC EV significantly decreased the therapeutic effects of HMW HA primed MSC EV on PA103 CFU and TNFα levels in the BALF and blood when compared with NC siRNA pretreated HMW HA primed MSC EV in mice. Data is median with IQR for PA103 CFU and mean ± SD for TNFα levels, *P < 0.01 by Mann Whitney U test or Student t-test, *N* = 6. (B) By histology, administration of HMW HA primed MSC EV pretreated with CD44 siRNA eliminated the therapeutic effects in terms of alveolar inflammatory cells infiltration, interstitial wall thickening, and blood/edema. Data is mean ± SD, ****P < 0.0001 by Student t-test, *N* = 20. A representative histology is shown. Magnification 4X, Bar = 1 mM. A dose of MSC EV (90 μl) was used = 2.0 × 10^10^ particles.

**Additional file 3: Supplementary Figure 3.** Trafficking of HMW HA (1.0 MDa) Primed MSC EV Pretreated with CD44 siRNA or NC siRNA in Mice with PA103 Bacterial Pneumonia. CD44 siRNA pretreatment of MSC EV significantly decreased the trafficking of HMW HA (1.0 MDa) primed MSC EV to the lung and liver. (A) Representative immunofluorescence from each organ with administration of HMW HA (1.0 MDa) primed MSC EV pretreated with CD44 siRNA or NC siRNA. Magnification 4X, Bar = 1 mM. (B) Quantification of the fluorescent intensity of MSC EV in the lung, liver, and spleen. Data is mean ± SD, **P < 0.01, ****P < 0.0001 by Student t-test, *N* = 12. A dose of MSC EV (90 μl) was used = 2.0 × 10^10^ particles.

## Data Availability

All the important data generated or analyzed in this study are included within this published article. Other data are available from the corresponding author on reasonable request.

## Abbreviations

ALI: Acute lung injury
HA: Hyaluronic acid
HMW HA: High molecular weight hyaluronic acid
LMW HA: Low molecular weight hyaluronic acid
MSC EV: Mesenchymal stem or stromal cell-derived extracellular vesicles
PA: *Pseudomonas aeruginosa*
siRNA: Small interfering RNA
