# Supplementary figures and images for: Role of CD44 in increasing the potency of mesenchymal stem cell extracellular vesicles by hyaluronic acid in severe pneumonia
Source: Stem Cell Res Ther. 2021 May 20;12:293. doi: 10.1186/s13287-021-02329-2 (PMC8136222; doi:10.1186/s13287-021-02329-2)

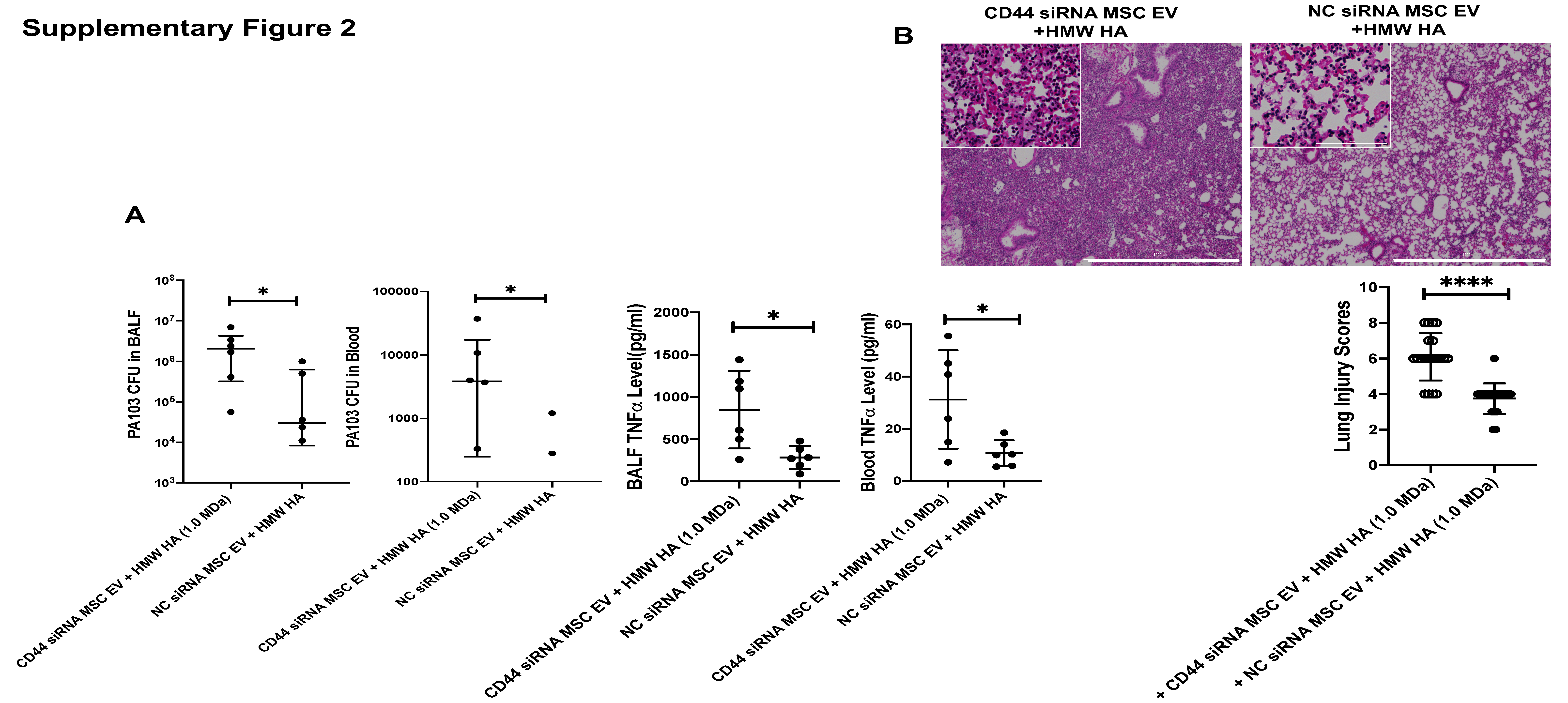

Supplement: Supplementary file 2 — Additional file 2: Supplementary Figure 2. Influence of CD44 siRNA Pretreatment of MSC EV in the Therapeutic Effects of HMW HA Primed MSC EV in Mice with PA103 Bacterial Pneumonia. (A) CD44 siRNA pretreatment of MSC EV significantly decreased the therapeutic effects of HMW HA primed MSC EV on PA103 CFU and TNFα levels in the BALF and blood when compared with NC siRNA pretreated HMW HA primed MSC EV in mice. Data is median with IQR for PA103 CFU and mean ± SD for TNFα levels, *P < 0.01 by Mann Whitney U test or Student t-test, N = 6. (B) By histology, administration of HMW HA primed MSC EV pretreated with CD44 siRNA eliminated the therapeutic effects in terms of alveolar inflammatory cells infiltration, interstitial wall thickening, and blood/edema. Data is mean ± SD, ****P < 0.0001 by Student t-test, N = 20. A representative histology is shown. Magnification 4X, Bar = 1 mM. A dose of MSC EV (90 μl) was used = 2.0 × 10^10^ particles. [file 13287_2021_2329_MOESM2_ESM.tiff]

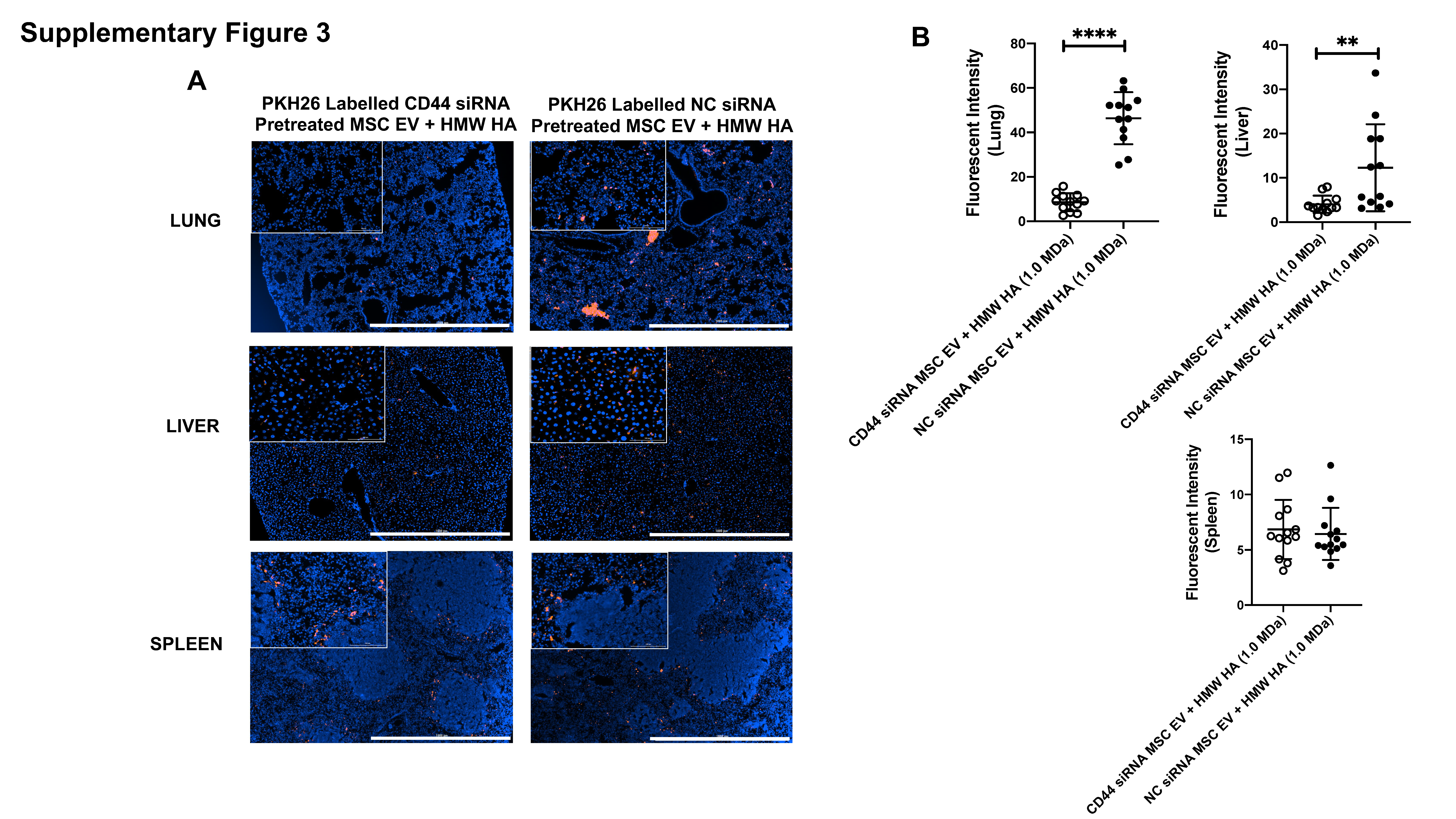

Supplement: Supplementary file 3 — Additional file 3: Supplementary Figure 3. Trafficking of HMW HA (1.0 MDa) Primed MSC EV Pretreated with CD44 siRNA or NC siRNA in Mice with PA103 Bacterial Pneumonia. CD44 siRNA pretreatment of MSC EV significantly decreased the trafficking of HMW HA (1.0 MDa) primed MSC EV to the lung and liver. (A) Representative immunofluorescence from each organ with administration of HMW HA (1.0 MDa) primed MSC EV pretreated with CD44 siRNA or NC siRNA. Magnification 4X, Bar = 1 mM. (B) Quantification of the fluorescent intensity of MSC EV in the lung, liver, and spleen. Data is mean ± SD, **P < 0.01, ****P < 0.0001 by Student t-test, N = 12. A dose of MSC EV (90 μl) was used = 2.0 × 10^10^ particles. [file 13287_2021_2329_MOESM3_ESM.tiff]
